# Could Azithromycin Be Part of *Pseudomonas aeruginosa* Acute Pneumonia Treatment?

**DOI:** 10.3389/fmicb.2021.642541

**Published:** 2021-03-16

**Authors:** Anne-Gaëlle Leroy, Jocelyne Caillon, Nathalie Caroff, Alexis Broquet, Stéphane Corvec, Karim Asehnoune, Antoine Roquilly, Lise Crémet

**Affiliations:** ^1^Laboratoire EA 3826 “Thérapeutiques cliniques et expérimentales des infections”, IRS2-Nantes Biotech, Université de Nantes, Nantes, France; ^2^CHU de Nantes, Service de Bactériologie-Hygiène hospitalière, Nantes Université, Nantes, France; ^3^CRCINA, U1232, CHU Nantes, Nantes, France; ^4^CHU de Nantes, Service Anesthésie Réanimation Chirurgicale, Nantes Université, Nantes, France

**Keywords:** macrolide, *Pseudomonas aeruginosa*, adjunctive therapy, critically ill patients, acute pulmonary infections, immunomodulation, ribosome, quorum sensing

## Abstract

Azithromycin (AZM) is a 15-membered-ring macrolide that presents a broad-spectrum antimicrobial activity against Gram-positive bacteria and atypical microorganisms but suffers from a poor diffusion across the outer-membrane of Gram-negative bacilli, including *Pseudomonas aeruginosa* (PA). However, AZM has demonstrated clinical benefits in patients suffering from chronic PA respiratory infections, especially cystic fibrosis patients. Since the rise of multidrug-resistant PA has led to a growing need for new therapeutic options, this macrolide has been proposed as an adjunctive therapy. Clinical trials assessing AZM in PA acute pneumonia are scarce. However, a careful examination of the available literature provides good rationales for its use in that context. In fact, 14- and 15-membered-ring macrolides have demonstrated immunomodulatory and immunosuppressive effects that could be of major interest in the management of acute illness. Furthermore, growing evidence supports a downregulation of PA virulence dependent on direct interaction with the ribosomes, and based on the modulation of several key regulators from the Quorum Sensing network. First highlighted *in vitro*, these interesting properties of AZM have subsequently been confirmed in the animal models. In this review, we systematically analyzed the literature regarding AZM immunomodulatory and anti-PA effects. *In vitro* and *in vivo* studies, as well as clinical trials were reviewed, looking for rationales for AZM use in PA acute pneumonia.

## Introduction

Originally isolated from *Streptomyces* species, macrolide antibiotics are composed of a central macrocyclic lactone ring of 14, 15, or 16 atoms of carbon to which various sugar substituents are attached (Retsema and Fu, 2001). Erythromycin, a 14-membered-ring molecule, was the first macrolide commercially available. To extend erythromycin’s spectrum of activity and to improve it’s pharmacokinetic and tolerance profiles, semisynthetic compounds have been subsequently developed, such as azithromycin (AZM), which differs from erythromycin by the inclusion of a methyl-substituted nitrogen in the macrolide ring (Retsema et al., 1987; Retsema and Fu, 2001). Indeed, this 15-membered-ring macrolide presents a broad-spectrum antimicrobial activity against Gram-positive bacteria, some atypical microorganisms like *Mycoplasma* spp. or *Chlamydia* spp., and some Gram-negative bacteria like *Haemophilus influenzae*, *Campylobacter* spp., *Neisseria gonorrhoeae*, or *Legionella pneumophila*. Due to a poor diffusion across the outer-membrane and an active efflux, *Pseudomonas aeruginosa* (PA), and members of the *Enterobacteriaceae* family are generally insensitive to AZM (Retsema et al., 1987; Nikaido, 1998; Masuda et al., 2000; Gomes et al., 2017). Nonetheless, this molecule is recommended as an alternative for the treatment of some diarrhoeagenic Enterobacteriaceae (*Salmonella* spp. or *Shigella* spp.; Lübbert, 2016), suggesting a better *in vivo* activity than expected.

Macrolides exert their bacteriostatic effects by inhibiting bacterial protein synthesis, thereby preventing bacterial multiplication. Through reversible interaction with the 23S rRNA, macrolides block the peptide exit channel of the 50S ribosomal subunit, inhibiting bacterial protein translation. In this way, they prevent the progression of the nascent chain, causing premature detachment of incomplete peptide chains. This phenomenon known as “peptidyl-tRNA drop-off” leads to the depletion of intracellular pools of aminoacyl-tRNA available for protein synthesis (Retsema and Fu, 2001; Lovmar et al., 2004, 2009; Starosta et al., 2010; Vázquez-Laslop and Mankin, 2018).

*Pseudomonas aeruginosa* is a Gram-negative opportunistic pathogen notably responsible for acute and chronic pulmonary infections, particularly in patients with compromised systemic immunity or impaired mucosal defenses, as in the case of ventilator-associated pneumonia (VAP) or cystic fibrosis (CF). PA is intrinsically resistant to a wide range of antibiotics and can develop resistance to multiple classes of antibiotics, leading to serious therapeutic challenges (Lister et al., 2009; De Oliveira et al., 2020). AZM has been suggested as a new adjunctive therapeutic option in PA treatment, while this species presents high minimum inhibitory concentrations (MIC) for that antibiotic (from 8 to 512 mg/L or higher; Howe and Spencer, 1997; Imperi et al., 2014). However, long-term low-dose AZM treatment has been reported to positively influence the clinical outcome in patients suffering from chronic PA infections like in diffuse panbronchiolitis or CF, suggesting anti-PA effects of AZM (Saiman et al., 2003; Hansen et al., 2005; Mayer-Hamblett et al., 2018). Besides, AZM has been shown to present beneficial effects in viral respiratory infections (influenza virus, respiratory syncytial virus, …) implying effects on the immune system (Kakeya et al., 2014; Beigelman et al., 2015). Additionally, pharmacokinetics studies have underlined the favorable profile of AZM with an excellent tissue penetration, particularly in the lungs, and a high intracellular accumulation, notably in white blood cells and alveolar macrophages (Baldwin et al., 1990; Conte et al., 1996; Lucchi et al., 2008; Matzneller et al., 2013), which makes AZM a candidate of particular interest for the treatment of respiratory infections.

In this review, we systematically analyzed literature regarding AZM anti-PA and immunomodulatory effects. *In vitro* and *in vivo* studies, as well as clinical trials were indiscriminately reviewed, looking for rationales for AZM use in PA acute pneumonia.

The impact of AZM as an additional treatment against PA chronic respiratory infections, including CF lung disease, as well as effects of AZM on biofilm formation will not be explored in this review. Indeed, these questions have already been well-studied and reviewed (Gaylor and Reilly, 2002; Wolter et al., 2002a; Tateda et al., 2007; Cai et al., 2011).

## Macrolides and AZM: From Antibiotic to Immunomodulatory Effects: What Do We Know?

In addition to their antibacterial activity, regulatory effects on the immune response have been attributed to macrolides soon after their introduction (Parfenova and Ryviakova, 1963; Gluzman, 1964; Plewig and Schöpf, 1975). Even if underlying mechanisms are not yet fully elucidated, growing evidence supports that macrolides, especially 14- and 15-membered ring compounds, would have anti-inflammatory and immunomodulatory properties.

**Figure 1:**
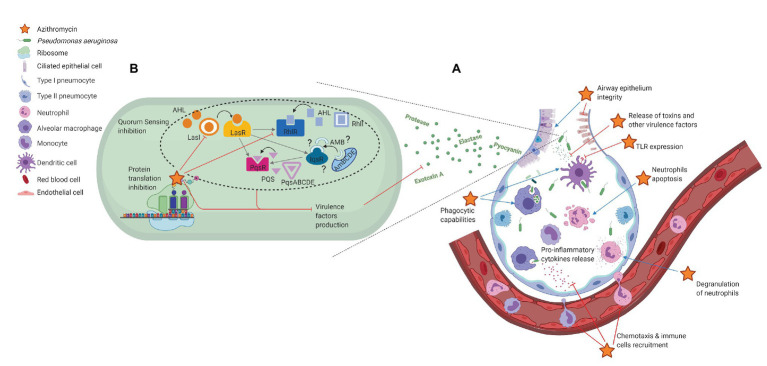
Non-antibiotic effects of azithromycin in *Pseudomonas aeruginosa* acute pneumonia. **(A)** Immunomodulatory effects; **(B)** *P. aeruginosa* virulence reduction. Effects of azithromycin are shown as inhibition (red) or stimulation (blue).

After an overview of the innate immune mechanisms involved in responses to PA pulmonary infections, this first part of the review will focus on *in vitro* studies that support a macrolide modulation of these immune mechanisms (Figure 1A).

### Innate Immune Responses to PA Pulmonary Infections

*Pseudomonas aeruginosa* virulence depends on an arsenal of cell-associated and extracellular factors that can damage the respiratory epithelium and alter innate and adaptative responses, thus allowing the establishment of severe lung infections (Curran et al., 2018). Innate immune responses play a critical role in controlling PA acute infections. Recognition of PA in the respiratory tract is mediated by pattern recognition receptors (PRRs) expressed on the pulmonary residential innate immune cells (alveolar macrophages, dendritic cells, airway epithelial cells, …), notably Toll-like receptors (TLR). TLR4 specifically recognizes the lipopolysaccharide (LPS) of the outer membrane of PA, while TLR5 interacts with the flagellum of the bacterium. This recognition induces the release of pro-inflammatory cytokines (TNF-α, IL-6, and IL-1β) and chemokines (IL-8) and elicits the massive recruitment of neutrophils from the peripheral blood. The infiltrated neutrophils play a primary role in PA clearance. However, their degranulation also contributes to inflammation and damages tissues, their presence in the airway remaining no longer beneficial once PA has been cleared (Lavoie et al., 2011; Curran et al., 2018; Kumar, 2020).

### Macrolides Enhance Airway Epithelial Integrity

Different macrolides, including AZM, were shown to stabilize human respiratory epithelium upon inflammatory *in vitro* conditions (Feldman et al., 1997). Beneficial effects of AZM on human airway epithelial integrity were then demonstrated by an increase of the transepithelial electrical resistance in human bronchial epithelial cells (Asgrimsson et al., 2006). These data were confirmed in a model of human airway epithelium infected with PA (Halldorsson et al., 2010), and later in a cell model mimicking ventilator-induced lung injury (Joelsson et al., 2020). The strengthening of the airway epithelial integrity contributes to the reduction of surrounding inflammation.

### Macrolides and the Innate Immune System

Numerous *in vitro* studies highlighted macrolides interactions with different stages of the innate immune response to infection. The main effects are summarized in the subsequent part of the review, following logical steps of the innate immune reaction.

#### Pathogen Recognition

Toll-like receptors recognition of conserved motifs expressed by bacteria such as LPS plays an important role in innate immunity triggering. Macrolides have been shown to reduce TLRs expression, in particular on monocytes (Park et al., 2005), macrophages (Nujić et al., 2012), and dendritic cells (Iwamoto et al., 2011). Different mechanisms have been suggested including an impairment of lysosomal functions leading to a deregulation of TLRs recycling (Nujić et al., 2012) and an inhibition of NF-κB expression (Iwamoto et al., 2011).

A dysregulated innate immune response during infection may increase its severity, notably by causing increased and irreversible organ damages (Kumar, 2020). Thus, reduction of TLRs surface expression and impairment of TLRs signaling contribute to the modulation of excessive inflammatory response, deleterious in critically ill patients.

#### Pro-inflammatory Mediators, Cytokines, and Chemokines Production

Pro-inflammatory mediators, cytokines, and chemokines are key regulators of the immune response that mediate cell-to-cell communication. Pro-inflammatory cytokines (e.g., IL-1, IL-6, INF-γ, and TNF-α) and chemokines (e.g., IL-8 and RANTES) magnify the immune response. Interestingly, macrolides seem to decrease excessive inflammatory response detrimental to the host. Indeed, 14- and 15-membered-ring macrolides, especially AZM, were shown to reduce, in a concentration-dependent manner and at physiologically achievable concentrations, the production of free oxygen radicals and numerous pro-inflammatory cytokines [e.g., TNF-α, IL-1, IL-6, IL-8, granulocyte-macrophage colony-stimulating factor (GM-CSF), …] by LPS-stimulated human monocytes and murine macrophages (Khan et al., 1999; Suzaki et al., 1999; Ianaro et al., 2000; Kikuchi et al., 2002). These observations are consistent with the reduction of TLRs expression already discussed. Besides, macrolides would inhibit IL-6, IL-8, and RANTES release from human bronchial epithelial cells *in vitro* (Takizawa et al., 1997; Yokota et al., 2012). In all, pro-inflammatory signals reduction would lead to a limitation of immune cell recruitment to the site of infection (Zimmermann et al., 2018). Moreover, Tsai et al. (2004) demonstrated an impairment of neutrophils chemotaxis in response to different chemotactic stimuli after AZM exposure.

#### Effects on Cell Functionalities

Polymorphonuclear neutrophils and phagocytic cells are key players in the elimination of microorganisms. Beyond macrolides’ effects on their recruitment, impacts on their functionalities are of particular interest. Hence, macrolides have been shown to stimulate degranulation of neutrophils, thus enhancing their antibacterial activity (Abdelghaffar et al., 1996). AZM has also been shown to improve phagocytic capabilities of the alveolar macrophages and dendritic cells (Hodge et al., 2006; Polancec et al., 2012; Lin et al., 2016), and to increase bacterial clearance thanks to a phagosomal stability enhancement in human lung macrophages (Persson et al., 2012).

#### Apoptosis

Macrolides have been reported to shorten neutrophils survival by an acceleration of apoptosis during *in vitro* experiments (Aoshiba et al., 1995; Inamura et al., 2000; Koch et al., 2000). As already assessed, cell death by apoptosis strongly minimizes inflammation and host tissue damage in comparison to necrosis (Savill, 1997). Thus, neutrophils apoptosis promotion by macrolides could be an effective mechanism to promote the resolution of inflammation when it is not beneficial anymore.

Overall, numerous *in vitro* studies have highlighted the immunomodulatory properties of AZM and other 14- and 15-membered macrolides that would help to optimize the innate immune response, enabling limited immune cells recruitment on the infective site, but also enhancing functionalities of the recruited cells against bacterial pathogens.

#### Macrolides Influence on Cell Signaling

Consistent data indicate that mechanisms underlying macrolides regulatory effects on the immune response, rely on the modulation of cell signaling pathways. Hence, modulation of Ca^2+^ signal pathways has been suggested in neutrophils (Mitsuyama et al., 1997) and in rat mast cells (Kase et al., 2009). Interactions with MAPK signaling pathways have also been identified. MAPKs (including ERK, JNK, and p38 MAPK) play essential roles in immune responses, notably in the regulation of inflammatory cytokines expression, cell proliferation, differentiation, and apoptosis (Zhang and Dong, 2005). For example, it has been shown that AZM modulates pro-inflammatory cytokines secretion in human bronchial epithelial cells in part through the ERK pathway (Shinkai et al., 2006). The production of mucin by epithelial cells in response to PA infection was also shown to be reduced by AZM through interference with ERK (Imamura et al., 2004). Besides, a significant impairment in chemotaxis of human neutrophils was observed after AZM exposure, and showed to be mediated *via* an inhibition of the ERK signal transduction pathway (Tsai et al., 2004). Other studies documented an inhibitory effect of macrolides on transcription factors, such as nuclear factor kappa B (NF-κB) and activator protein-1 (AP-1; Desaki et al., 2000; Kikuchi et al., 2002). Interactions with cell signaling, in particular, MAPK signaling and transcription factors expression and function, may explain most of the immunomodulatory effects attributed to macrolides.

Hence, strong *in vitro* evidence underlines macrolides’ immunomodulatory effects that may play a beneficial role in PA respiratory infections. However, some direct effects on the bacterium should not be excluded without further investigations.

## *In Vitro* Anti-*Pseudomonas* Activity of Azithromycin

Due to membrane impermeability and active efflux pumps, PA presents high MICs to AZM (8 to >512 mg/L) and is considered insensitive to this antibiotic (Nikaido, 1998; Masuda et al., 2000; Imperi et al., 2014). However, AZM has been reported to improve clinical outcomes of PA infected patients (Howe and Spencer, 1997; Imperi et al., 2014), despite clinically-achievable concentrations in the infective sites far below these MICs [AZM concentrations do not exceed ~3 mg/L in epithelial lining fluid (ELF), according to the study of Lucchi et al. (2008)]. Thus, a reassessment of the assumed *in vitro* inactivity of AZM against PA and an exploration of the ways by which AZM may exhibit therapeutic activity is required.

### AZM Reduces the Expression of PA Virulence Factors

Shortly after its introduction in human medicine, some *in vitro* studies have demonstrated that AZM could reduce the activity of different exoenzymes (such as elastase, protease, exotoxin A, lecithinase, DNase, phospholipase C, but also haemolysin and gelatinase to a lesser degree) in PA isolates at concentrations below their MICs (sub-MICs) (Molinari et al., 1992, 1993; Mizukane et al., 1994). The same authors also highlighted the ability of AZM to interfere with pyocyanin production, motility, and flagellar synthesis (Molinari et al., 1992, 1993). In these studies, AZM exhibited the strongest virulence-suppressing effect among the different macrolides tested. This aptitude to disturb several pathogenic properties of PA *in vitro* rightly gave rise to the hope of a substantial reduction of PA virulence *in vivo*. However, the underlying mechanisms were at that time still unknown.

### PA Quorum-Sensing Network

Since these first experiments published by Molinari and Mizukane, the key role of a cell density-based intercellular communication network, widely known as the quorum-sensing (QS) system, in the regulation of virulence genes expression in PA has been established. *Pseudomonas aeruginosa* QS network relies on three interconnected systems: two acyl-homoserine lactones (AHL) QS systems, LasI-LasR and RhlI-RhlR, and a third system relying on the production of alkyl-quinolones (AQ) named the *Pseudomonas* quinolone signal (PQS) system (Jimenez et al., 2012; García-Reyes et al., 2020). Basically, each of these three QS systems is composed of a gene encoding a transcriptional regulator (*lasR*, *rhlR*, and *pqsR*), and a gene encoding an autoinducer synthetase (*lasI*, *rhlI*, and *pqsABCDE*, respectively) required for the synthesis of the autoinducer molecules, AHL and AQ. Recently, a fourth QS system has been suggested and named integrated QS signal (IQS; Lee et al., 2013; Lee and Zhang, 2015), but the existence of this system remains controversial as pointed out by Cornelis et al. (2020). These QS systems are hierarchically connected, and the Las system is at the top of the signaling hierarchy (Jimenez et al., 2012; Lee and Zhang, 2015). Depending on cell density, QS is responsible for the regulation of around 10% of PA genes including *lasA*, *lasB*, *toxA*, the *phzA1-phzG1*, and *phzA2-phzG2* operons, and *phzM* encoding for elastases, exotoxin A, and pyocyanin synthesis, respectively (Williams and Cámara, 2009; Jimenez et al., 2012; Lee and Zhang, 2015). Moreover, recently, high throughput studies revealed complex functional crosstalk between several key virulence-associated transcription factors considered as master regulators (such as PhoB, AlgR, ExsA, or GacA) and QS, in response to environmental changes (Huang et al., 2019). Since several QS related virulence factors are downregulated by AZM, the interference of AZM with the QS circuitry has been considered.

### AZM Reduces QS Autoinducer Molecules Production in PA

Tateda et al. (2001) highlighted that AZM could simultaneously reduce the expression of *lasR*, *rhlR*, *lasI*, and *rhlI*, leading to a decrease in AHL concentrations. Using a DNA microarray approach, Kai et al. (2009) pointed out a significant inhibition of the S-adenosyl methionine (AHL ultimate precursor) synthesis pathway, suggesting a reduction in autoinducers production under AZM conditions. Later, in 2016, the effects of AZM on QS signals were confirmed by Zeng and colleagues. Using HPLC-MS/MS, they demonstrated a significant decline in AHL synthesis when PA was cocultured with AZM. A transcriptional analysis also established a reduction in *lasI* expression in the presence of AZM (Zeng et al., 2016). Moreover, by another approach, Pérez-Martínez et al. demonstrated in 2011 that AZM reduces the expression of several genes from the Gac/Rsm signal transduction pathway, which positively controls the QS machinery in PA (Kai et al., 2009; Pérez-Martínez and Haas, 2011; Imperi et al., 2014). Collectively, these results are consistent with a direct or indirect inhibition of QS signals and make conceivable the modulation of several QS-activated virulence factors by AZM.

### AZM Downregulates QS-Dependent Genes Expression

A global analysis of the transcriptome and proteome profiles of PA exposed to AZM sub-MICs led to the identification of a large common subset of QS- and AZM-regulated genes/proteins (Nalca et al., 2006). In accordance with QS-dependent genes impeding, this study demonstrated (i) a downregulation of a wide range of virulence factors, (ii) an oxidative stress response impairment, (iii) an altered motility, and (iv) a strong induction of the type III secretion system (previously shown to be negatively regulated by QS; Hogardt et al., 2004; Nalca et al., 2006). Further studies, based on DNA microarray analysis of gene expression in the presence of AZM supported the previous findings, namely a potent alteration of the expression of a range of QS-regulated virulence factors (e.g., exotoxin A, elastase, alkaline metalloproteinase, …), and an induction of the expression of genes related to type III secretion system (Skindersoe et al., 2008; Kai et al., 2009). To go further, Zeng et al. (2016) suggested that AZM virulence-suppressing effect is time-dependent. Indeed, the authors showed that the addition of AZM resulted in decreased AHL production but increased the expression of selected genes regulated by LasR (*aprX* and *lasA*), RhlR (*rhlA* and *rhlB*), or PQS (*phnA* and *phnB*) during the exponential phase of growth. These results are in accordance with the fact that in the QS network, each system may take over the functions of the others, the three regulons acting in concert to control the timing of virulence genes expression at the optimal cell density. In the study of Swatton et al. (2016) comparison of the proteomes of wild-type PA and isogenic QS mutants exposed to AZM, confirmed that AZM influences the expression of some QS-regulated proteins. However, only a small fraction of the QS regulon was affected by AZM, and no more than half of the changes occurred within QS-regulated proteins. These data suggested that AZM also interferes with virulence *via* QS-independent mechanisms. This assumption was strengthened by the demonstration of an AZM virulence attenuation in a specific *Serratia marcescens* strain, lacking a functional QS system.

Collectively, these studies undeniably demonstrate that AZM negatively interacts with QS-regulated virulence factors. However, molecular mechanisms underlying these effects remain elusive and QS-independent processes are highly likely.

### Even in PA, the Target Remains the Ribosomes

The transcriptomic analysis conducted by Nalca et al. (2006) revealed that genes encoding ribosomal subunits, initiation, and elongation factors were overexpressed in AZM-treated PA. These results suggested that these genes may be overexpressed to compensate for impaired transpeptidation and/or translation due to AZM exposition. They implied that in PA, like in Gram-positive bacteria, AZM would bind to the 23S rRNA of the 50S ribosomal subunit, leading to an inhibition of bacterial protein translation (Skindersoe et al., 2008). Consistent with these findings, ribosome protection from AZM binding (by expressing the 23S rRNA methylases ErmBP from *Clostridium perfringens* or ErmC from *Staphylococcus aureus*, in PA), prevented AZM’s interference on elastase and rhamnolipid production, swarming motility, and biofilm production (Köhler et al., 2007; Glansdorp et al., 2008). Furthermore, the use of biotin-tagged azithromycin analogs in pull-down assays demonstrated that ribosomal-related proteins were the main binding partners of AZM in PA (Glansdorp et al., 2008). In addition, the discovery of specific mutations in domain V of the 23S rRNA in clinical PA isolates from CF patients also reinforced the hypothesis that AZM effects are mediated through a ribosome binding. Indeed, the observed mutations had already been reported to confer macrolides resistance in other bacterial species, and they reduced inhibition of growth by AZM at 50 μg/ml (Marvig et al., 2012). Recently, a *msr(E)* exogenous expression was shown to protect laboratory strains of PA from AZM downregulation of QS-regulated virulence factors (namely rhamnolipid and elastase production, and swarming motility). Besides, transcriptomes of PA strains expressing *msr(E)* were almost unaffected by AZM exposition (Ding et al., 2018). Msr(E) is a member of the ABC-F proteins, that were shown to displace antibiotics from ribosomes *in vitro*, rescuing translation from antibiotic-mediated inhibition (Sharkey et al., 2016). Taken together, these data indicate that the impact of AZM on virulence factors production in PA is dependent upon binding to the ribosomes.

**Figure 2:**
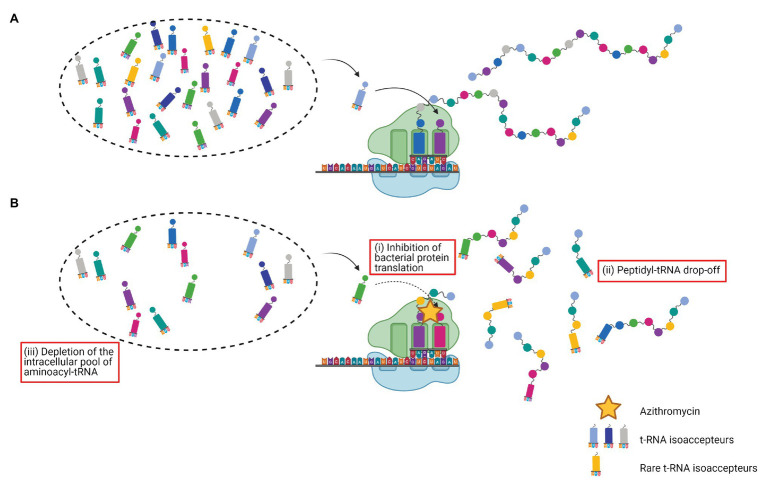
Effects of azithromycin on the peptidyl-tRNA drop-off and on the depletion of rare tRNAs isoacceptors. **(A)** Protein synthesis; **(B)** azithromycin (i) blocks the peptide exit channel of the 50S ribosomal subunit, inhibits bacterial protein translation, and causes premature detachment of incomplete peptide chains, (ii) leading to an increased peptidyl-tRNA drop-off, and (iii) resulting in a depletion of the intracellular pool of aminoacyl-tRNA. Rare tRNAs isoacceptors are the most affected.

To go further, it has been suggested that bacterial protein translation inhibition by AZM leads to an increased peptidyl-tRNA drop-off, resulting in a depletion of the intracellular pool of aminoacyl-tRNA (Lovmar et al., 2004, 2009). By overexpressing a peptidyl-tRNA hydrolase Pth (a specific enzyme responsible for the release of tRNA from the peptidyl-tRNA, thereby increasing the pool of free tRNA available for protein synthesis; Kössel and RajBhandary, 1968), Gödeke et al. (2013) elegantly demonstrated the critical role of tRNA pool depletion in the AZM’s virulence downregulation effect. As expected, overexpression of Pth in a PA strain increased the fraction of uncharged tRNAs, counteracted the AZM effects on rhamnolipid and pyocyanin production, partially restored the swarming motility, and reduced PA cytotoxicity over A549 cells. Besides, the authors brought an attractive rationale for the previously discussed selective activity of AZM on a subset of proteins. Indeed, they showed that AZM preferentially decreases availability of tRNAs isoacceptors that bind to rare codons. Thus, interestingly, the exchange of the rarely used second codon of the *rhlR* gene by a more frequently used, drastically reduced AZM downregulation of rhamnolipid and pyocyanin production. Hence, they demonstrated that *rhlR* belongs to the highly AZM affected genes because of the presence of rare codons in its sequence (Gödeke et al., 2013). The effects of AZM on the peptidyl-tRNA drop-off and on the depletion of rare tRNAs isoacceptors in the intracellular pool are presented in Figure 2.

### Could AZM Exert Direct Antimicrobial Activity Against PA?

A bactericidal effect of AZM on stationary-phase PA cells has been suggested (Tateda et al., 1996, 2000; Imamura et al., 2005) without QS modulation being an acceptable explanation. Thus, Imamura et al. (2005) proposed a destabilization of the outer-membrane, but, according to other reports, the killing effect of AZM on PA at the stationary growth phase would more likely rely on the protein machinery inhibition. Indeed, ribosome protection drastically inhibited this effect (Köhler et al., 2007; Marvig et al., 2012; Gödeke et al., 2013).

An impact of culture media on PA susceptibility to macrolides has also been suggested. Indeed, an enhanced outer-membrane permeability combined with an impairment of active afflux mediated by oprM have been demonstrated in eukaryotic environments, and would result in increased susceptibility of PA to AZM, when cultured in eukaryotic media as compared to conventional broths (Buyck et al., 2012; Meerwein et al., 2020). These data may help explain a clinical efficacy of macrolides against PA infections that would not be predicted with conventional antibiotic susceptibility testing systems.

Overall, these numerous studies demonstrated that (i) even in PA, AZM interacts with ribosomes and affects protein synthesis, (ii) AZM inhibits protein synthesis to different extents, depending on the number of rare codons within nucleotide sequences, (iii) QS components may be part of the most affected genes, and (iv) QS inhibition modulates the expression of numerous QS-dependent virulence factors. The effects of AZM on PA virulence are schematically summarized in Figure 1B.

These *in vitro* data are expanding and could point to a clinical benefit of AZM use in the management of PA infections. AZM’s inhibition of PA QS is of particular interest given the importance of QS systems in PA pathogenesis in several animal studies (Rumbaugh et al., 1999; Lesprit et al., 2003). However, these *in vitro* analyses might also be too simplistic, notably because they are performed under perfectly controlled conditions, and because AZM concentrations employed are mostly higher than achievable concentrations in infective sites (Lucchi et al., 2008). A careful analysis of AZM evaluation in the animal models is also essential.

## Efficacy of Macrolides Combined Therapy in Animal Models of Acute *Pseudomonas Aeruginosa* Pneumonia

The first investigation of the influence of adjunctive AZM in the treatment of PA murine acute pneumonia dates back to 1999. Using a mucoid PA strain, the authors obtained significant improvements in mice survival when AZM was combined with ceftazidime (CAZ), compared to CAZ alone. The addition of AZM did not reduce bacterial loads within the lungs and the way AZM improved the outcome remained unclear (Nicolau et al., 1999). This first study, however, paved the way for numerous animal studies.

### AZM Attenuates Airway Inflammation

As previously reviewed, macrolides, and specifically AZM, have demonstrated anti-inflammatory properties throughout *in vitro* studies. These properties were confirmed *in vivo* and appeared beneficial in the management of the deleterious inflammatory process consecutive to acute bacterial pneumonia.

#### AZM Exerts Anti-inflammatory Properties in Nonbacterial Models

Regarding noninfectious models, AZM demonstrated effective anti-inflammatory properties in a mouse model of allergic asthma. Hence, three days after an allergen respiratory challenge, levels of total immune cells (including neutrophils, eosinophils, lymphocytes, and macrophages) and inflammatory mediators (in particular IL-5, and IL-13, known to mediate allergic airway inflammation), were reduced in lung tissues and bronchoalveolar lavage fluids (BALF) in AZM-treated mice. Irrespective of the timing of AZM subcutaneous administrations (before, throughout, or after the allergen challenge), AZM mediated attenuation of the airway inflammation (Beigelman et al., 2009). In a mouse model of paramyxoviral bronchiolitis, AZM significantly attenuated total leukocytes, and accumulation of inflammatory mediators in lungs and BALF, without changing viral loads. This airway inflammation reduction also contributed to a better tolerance of the infection, illustrated by an attenuated post-viral weight loss (Beigelman et al., 2010). Furthermore, in a murine model of LPS-induced pulmonary neutrophilia (Szarka et al., 1997), prophylactic intraperitoneal administration of AZM allowed a significant reduction of the total number of cells, as well as neutrophils percentage in BALF, in comparison to untreated mice. Later, AZM was also shown to reduce BALF concentrations of pro-inflammatory cytokines [TNF-α, IL-6, and macrophage inflammatory protein 2 (MIP-2)] in the same LPS model (Ivetić Tkalcević et al., 2006). As the LPS-induced pulmonary neutrophilia model is acknowledged to mimic acute respiratory distress syndrome secondary to bacterial pneumonia, these works suggested beneficial effects of AZM in acute bacterial pneumonia management. The noninfectious models are of particular interest because they allow distinguishing anti-inflammatory properties from virulence reduction. Here, they laid the foundation for an AZM-mediated airway inflammation reduction.

#### AZM Reduces Inflammation in Non-PA Acute Bacterial Pneumonia

Yamada et al. (2013) reported that AZM monotherapy drastically improved survival rates in a mouse model of ventilator-associated pneumonia caused by a multidrug-resistant *Acinetobacter baumannii*, despite no antimicrobial effect (confirmed by examination of bacterial loads in lungs). Lungs histopathological analysis and BALF cell counts revealed a decline in inflammatory cells infiltration, notably neutrophils, in AZM-treated mice compared to the control group. Pro-inflammatory cytokines levels (namely IL-1β, IL-6, and MIP-2) were also significantly lower in BALF when AZM was administrated. An AZM modulation of the host inflammatory response was also demonstrated in a mouse model of AZM-resistant pneumococcal lethal pneumonia (Yoshioka et al., 2016). The adjunction of AZM to ceftriaxone led to a survival benefit despite similar bacterial burdens in the lungs. The influx of cells, especially neutrophils, in lungs was significantly lower in the ceftriaxone-plus-azithromycin group, suggesting a modulation of a potentially excessive host inflammatory response. Moreover, the analysis of cell surface markers demonstrated that AZM reduces the immunosuppressive state following sepsis through a downregulation of coinhibitory molecules [namely cytotoxic-T lymphocyte-associated antigen 4 (CTLA-4) and programmed death 1 (PD-1)]. Taken together, these data suggested a real enhancement of the innate immunity efficiency by AZM, both by reducing the excessive inflammatory response, and by restoring immune functions after sepsis (Yoshioka et al., 2016).

#### AZM Reduces Inflammation in PA Pneumonia

*In vivo* studies assessing the anti-inflammatory effects of AZM in PA acute pneumonia are scarce, but multiple lines of evidence suggesting clinical improvements in CF patients under long-term macrolides treatment (Jaffé et al., 1998; Wolter et al., 2002b; Saiman et al., 2003) led to experiments using chronic models of pneumonia.

A chronic model of endobronchial infection (PA embedded in agar beads) was used to assess the effects of AZM on bacterial burden, inflammatory cell influx, and cytokines production within the lungs of mice (Tsai et al., 2004). While pulmonary clearance of PA was not affected by AZM treatment, administration of the macrolide resulted in a significant decrease in the recruitment of inflammatory cells, notably neutrophils, to the airway. Impairment in neutrophils recruitment was both explained by a lung level reduction of proinflammatory cytokines (namely TNFα and KC) and by an impressive attenuation of neutrophil chemotactic responses to stimuli *in vitro*. The observed effects on chemotaxis were also noted with human neutrophils, after IL-8 stimulation *in vitro*. A few years later, using a CF murine model of PA pneumonia, Tsai et al. (2009) confirmed that AZM reduces lung inflammation (again, both inflammatory cells and cytokines were decreased). In this model, a significant reduction in lungs bacterial loads was observed, and was attributed to an enhancement of bacterial clearance, mediated by AZM. However, the authors did not provide any arguments to consolidate that theory, and an effect of AZM on PA virulence was not assessed. Moreover, in that study, AZM was shown for the first time to improve the clearance of apoptotic neutrophils by macrophages, which could probably help to reduce inflammation. Taken together, these two studies, conducted by the same researchers, strengthened the idea that AZM reduces pulmonary inflammation without affecting pulmonary clearance of PA. However, generalization of these results to PA acute pneumonia may be inappropriate.

We found only one study that specifically assessed the effects of a modified macrolide, EM703, lacking antibiotic activity, as an adjunct anti-inflammatory treatment in association with levofloxacin, in a PA acute pneumonia murine model (Kasetty et al., 2017). Interestingly, all mice receiving levofloxacin 4 h after infection died within 72 h, whereas no bacteria were detected in their lungs 48 h after infection. The fatal outcome was thus not related to a lack of bacterial eradication, suggesting the involvement of a dysregulated host response. Pretreatment (24 h before infection) and simultaneous administration (simultaneously with infection) of EM703 drastically improved survival, reduced lung levels of proinflammatory cytokines (IL-6, MCP-1, KC, and TNFα), and decreased lungs damages and neutrophils infiltration in EM703 and levofloxacin treated mice.

Taken together, these experimental results strongly underlined the beneficial anti-inflammatory properties of AZM as an adjuvant treatment in both infectious (including PA infections) and noninfectious mouse airway inflammation models. AZM’s management of an exaggerated, dysregulated, and deleterious host response to bacterial infection may prevent a progression toward acute lung injury. If AZM does not seem to directly reduce bacterial burden, modulation of bacterial virulence without impairing bacterial viability has never been assessed in the previously discussed studies. As several *in vitro* experiments have demonstrated a significant reduction of PA virulence by AZM, it will be interesting in future studies to investigate the *in vivo* effects of AZM on PA virulence.

### Effects of AZM on PA Virulence *in vivo*

Even though the effect of AZM administration in mice on PA virulence has never directly been assessed, two *in vivo* studies evaluated the virulence of PA following an exposure to AZM *in vitro*.

Firstly, Kobayashi et al. (2002) studied the virulence of a macrolide pre-treated PA in a murine model of acute pneumonia. Briefly, mice were challenged intranasally with PA pre-cultured for 24 h on Mueller-Hinton agar containing macrolides (notably AZM) at sub-MICs. Paradoxically, clarithromycin-, erythromycin-, and AZM-treated PA were more virulent than non-treated bacteria or bacteria treated with other antibiotics (ceftazidime, ofloxacin, or aminoglycosides). Indeed, the mortality rate of mice infected with AZM-treated PA achieved 100%, whereas none of the mice inoculated with non-treated PA died. Interestingly, more than half of the mice died within 9 h after the challenge with PA. The shortness of timing seemed to be more consistent with an acute toxic effect. Indeed, pulmonary permeability (measured by the wet weight of lungs and the total protein content in BALF) was drastically increased in mice infected by clarithromycin-treated PA. TNFα, NO, and elastase levels in BALF were also significantly increased in mice inoculated with clarithromycin-treated PA. Taken together, the overproduction of NO and TNFα, as well as the larger amount of elastase, may have accelerated inflammation and tissue damages in the lungs of mice challenged with macrolide-treated PA. Hence, surprisingly, whereas numerous *in vitro* studies have demonstrated that macrolides reduce PA virulence, the first study assessing the impact of macrolides pre-treatment on PA virulence *in vivo* rather demonstrated a virulence enhancement, with no obvious explanation.

In the second study, Stellari et al. (2015) used an IL-8/luciferase transgenic mouse model, for the *in vivo* monitoring of the IL-8 mediated lung inflammation induced by inoculation of culture supernatants from two PA CF clinical strains grown with AZM or not. Consistent with previous studies already discussed (Molinari et al., 1992, 1993; Mizukane et al., 1994), growth of one of both strains with AZM at sub-MIC inhibited the synthesis and release of various virulence factors (metalloprotease, pyocyanin, pyoverdine, motility, and biofilm formation). Twenty-four hours after mice stimulation with the corresponding supernatant, lung inflammation was significantly reduced, cellular infiltration (white blood cells and neutrophils) and pro-inflammatory cytokines levels [IL-1β, IL-17, RANTES, KC, and IL-12 (p70)] in BALF significantly decreased in comparison with animals challenged with untreated supernatant.

The key difference between these two studies concerns the nature of the products used to challenge mice: living PA in the first study vs. bacterial cells free supernatants in the second one. In the first study, mice did not receive antibiotics, and a rebound effect after PA introduction in the antibiotic-free airways might be one explanation for the virulence enhancement observed. As an AZM-mediated PA virulence downregulation might be a promising therapeutic approach, further experimental murine models assessing the effect of AZM administration on PA virulence are strongly needed. A clinical benefit associated with AZM treatment in PA acute pneumonia should be considered, at least through direct anti-inflammatory properties and certainly through an assumed PA virulence modulation. Existing experimental data provide a rationale for prospective randomized clinical trials.

## Human Clinical Trials

First shown to alter the natural history of diffuse panbronchiolitis (DPB), long-term macrolides treatment, including AZM, has since then consistently demonstrated clinical benefits (e.g., weight increase, reduction in the risk of pulmonary exacerbation, slowdown in lung function decline, as well as less colonization/infection with mucoid strains of PA) in patients suffering from chronic respiratory diseases [in particular CF, DPB, and chronic obstructive pulmonary disease (COPD)] (Jaffé et al., 1998; Wolter et al., 2002b; Saiman et al., 2003; Hansen et al., 2005; Spagnolo et al., 2013; Mayer-Hamblett et al., 2018). Pursuing the goal of this review, clinical trials assessing the effects of AZM in acute pneumonia are presented and discussed in the following section. Clinical studies involving PA acute pneumonia were of particular interest.

### Macrolide Adjunctive Therapy May Be Beneficial for the Management of Community-Acquired Pneumonia

A retrospective cohort study examined the impact of macrolides adjunctive therapy at the time of hospital admission on mortality of patients with severe sepsis due to CAP (Restrepo et al., 2009). The use of macrolides (erythromycin, clarithromycin, or AZM) was associated with decreased mortality at 30 and 90 days. The survival benefit remained after evaluating cases with macrolide-resistant pathogens. According to the authors, better outcomes associated with macrolide use would have resulted from an atypical pathogen coverage and/or from immunomodulatory effects. In another large cohort of patients with bacteremic CAP, the retrospective analysis of the patients’ outcomes found lower mortality in those treated by macrolides, whereas the use of fluoroquinolones or tetracyclines (also covering atypical pathogen) was not beneficial (Metersky et al., 2007). A prospective study performed on patients presenting an inadequate response after 72 h of antibiotic treatment for CAP, highlighted the benefits of an AZM adjunctive therapy associated with β-lactams on clinical outcomes and inflammatory responses (lower IL-6 and TNF-α in BALF, and IL-8 and IL-10 in blood) (Lorenzo et al., 2015). Among the 52 patients included, only seven suffered from PA pneumonia, making a sub-analysis on PA infections impossible. Interestingly, no beneficial effect was observed in the CAP control group (patients who reached clinical stability after 72 h of antibiotic treatment). In this study, the immunomodulatory effects of macrolides would have helped to resolve the excessive and inadequate inflammation observed in antibiotic treatment non-responders.

These encouraging results were undermined by a retrospective study focusing on PA CAP (Laserna et al., 2014). In this large cohort study, macrolide (erythromycin, clarithromycin, or azithromycin) therapy within the first 48 h of admission was not associated with lower 30-day mortality, ICU admission, need for mechanical ventilation, or length of stay in hospitalized patients with PA CAP. Even if the retrospective design of the study led to some heterogeneity in antipseudomonal antibiotics received by the patients, no significant difference was found in antibiotic coverage against PA in the first 48 h, between patients treated or not with a macrolide. Of note, only a third of the included patients needed an ICU hospitalization.

Taken all together, these studies suggested that (i) macrolides, including AZM, may be a beneficial adjunctive therapy during CAP, even when macrolide-resistant pathogens are involved, (ii) beneficial effects mostly rely on immunomodulatory effects, and (iii) beneficial effects seem higher among critically ill patients, also suffering from a stronger inflammatory response and immune dysregulation.

### Benefits of Macrolides in Critically Ill Patients With Acute Respiratory Failure

Secondary data analysis from the acute respiratory distress syndrome (ARDS) clinical trials network showed that the use of macrolides (erythromycin or AZM) within the first 60 h of acute lung injury (ALI) was associated with improved clinical outcomes, when compared with patients who did not receive macrolides. Indeed, a pronounced survival advantage and a shorter time to successful discontinuation of mechanical ventilation were observed in the macrolide treated group, regardless of the cause of the ALI (Walkey and Wiener, 2012). Beneficial effects of adjunctive therapy with AZM on ARDS of any etiology were confirmed with Kawamura et al. (2018). Again, significant improvement in the 90-day survival rate, and a shorter time to successful discontinuation of mechanical ventilation was observed. Concomitantly, low-dose macrolide (mainly erythromycin) therapy was associated with reduced 30-day mortality in a large cohort of patients with ARDS (Simonis et al., 2018). Authors of the above studies agreed with favorable immunological effects rather than antimicrobial effects of macrolides during ARDS.

In a randomized controlled trial, Giamarellos-Bourboulis et al. (2008) evaluated the immunomodulatory effects of clarithromycin in patients with sepsis and ventilator-associated pneumonia (VAP). The mortality rate at day 28 was not affected by clarithromycin treatment. However, clarithromycin significantly reduced the median times for resolution of VAP and for weaning from mechanical ventilation. In addition, the risk of death from septic shock and multiple organ dysfunctions (MODS) was significantly decreased in clarithromycin-treated patients. Thus, consistently with previous observations and with immunomodulatory effects, clarithromycin had the greatest effects on the most critically ill patients. Beneficial effects of clarithromycin could not be attributed to conventional antibiotic effects because Gram-negative pathogens were responsible for all documented infections (identification of the underlying pathogen was achieved in 70% of patients). A secondary analysis of the markers of inflammation in the patients included in this trial, suggested that clarithromycin reverses immune suppression and restores the balance between pro-inflammatory vs. anti-inflammatory mediators when severe sepsis and MODS develop (Spyridaki et al., 2012). Indeed, on day 4 after initiation of clarithromycin treatment, the analysis revealed a significant decrease of the serum IL-10/TNF-α ratio in clarithromycin-treated patients with septic shock and MODS. The expression of the co-stimulatory molecule CD86 on monocytes was also significantly increased on day 4, suggesting a better antigen presentation. All together, these data pointed toward a reversal of sepsis-induced immune suppression by clarithromycin. This hypothesis was strengthened by a subsequent analysis of the long-term mortality of these patients: 90-day mortality was significantly reduced in the clarithromycin group (Tsaganos et al., 2016).

Taken together, the successive analyses of this trial and the numerous studies conducted in patients with ARDS provided evidence that macrolides may adjust immune dysregulation in patients who are critically ill.

### AZM as an Anti-virulence Therapy to Prevent PA VAP

As already discussed, *in vitro* studies have demonstrated AZM interference with PA QS and virulence. AZM represents indeed an attractive anti-virulence therapy in PA infections and has been assessed in this indication in a few clinical trials.

*In vivo* anti-QS properties of AZM have mainly been evaluated in intubated patients colonized with PA (Köhler et al., 2010; van Delden et al., 2012). In these patients, according to the study of Köhler et al. (2010) AZM significantly reduced the expression of both QS-circuit (*lasI*) and QS target genes (*rhlA*), while an independent QS gene (*trpD*) was not affected. Interestingly, the authors found QS deficient *lasR* mutants in many patients. Furthermore, and maybe counterintuitively at first sight, whereas the proportion of *lasR* mutants significantly increased through time in the placebo group, *lasR* mutants slightly decreased in frequency in the AZM treated patients. Growing evidence suggests that in PA populations, *lasR* mutants are social cheaters that avoid the cost of producing QS-controlled factors while taking advantage of their production by the group (Diggle et al., 2007; Sandoz et al., 2007). These less virulent mutants may have a selective advantage in the presence of QS-wild-type PA, and their density would increase naturally during colonization (Köhler et al., 2009). In the above study, Köhler et al. (2010) highlighted that AZM therapy, by blocking QS in QS-wild-type isolates, may suppress the selective advantage of QS-mutants, and may increase the prevalence of more virulent QS-wild type isolates over the long term.

The study was not sufficiently powered to detect a significant effect of AZM on the occurrence of PA VAP, but a trend toward a reduced incidence of VAP was noted in the AZM-treated patients (van Delden et al., 2012). When analyzing the sub-group of patients colonized by fully QS-proficient isolates and thus, at highest risk for developing QS-related VAP (five AZM-treated vs. five placebo patients), the incidence of VAP was reduced 5-fold in the AZM group. Nevertheless, in this study, AZM efficacy to prevent PA VAP in colonized patients, was of less clinical benefit than initially hypothesized, certainly because most of the patients were colonized with PA QS-mutants (although the same workers also previously suggested that the proportion of QS-mutants would decrease over AZM treatment; Köhler et al., 2010).

In conclusion, this first clinical evaluation of anti-virulence effects of AZM for the prevention of PA VAP brought a proof of concept, but also underlined that further studies are needed to explore the consequences of this therapy on QS-deficient populations and to confirm an *in vivo* virulence reduction, but also to demonstrate a beneficial preventive effect on PA VAP. Until now, anti-virulence effects of AZM have never been evaluated in acute PA pneumonia.

Taken together, these clinical trials suggested that (i) AZM may be a beneficial adjunctive therapy during acute pneumonia, even when macrolide-resistant pathogens are involved, (ii) AZM beneficial effects strongly rely on immunomodulatory effects and are higher among critically ill patients, and (iii) AZM inhibition of the PA QS circuit may not be effective in mechanically ventilated patients because QS-deficient PA isolates appear to be common in that context. However, data on the reduction of PA virulence during human infections are scarce and are missing in PA acute pneumonia. Cautionary findings have been reported in some animal studies (Kobayashi et al., 2002), and some human clinical trials found no benefit of a macrolide therapy (Laserna et al., 2014). Thus, further prospective randomized controlled trials are strongly needed.

## Discussion

The data discussed reveal that AZM, by interfering with immune cells signaling pathways, modulates different stages of the immune response, and exerts interesting anti-inflammatory and immunomodulatory properties. Besides, this macrolide adjusts the expression of numerous PA virulence factors, through ribosome binding and interaction with PA QS network. These non-antibiotic effects, summarized schematically in Figure 1, are making AZM a good candidate for the management of PA acute pneumonia. However, this literature review also underlines that further *in vivo* experiments and prospective controlled trials are required to confirm the beneficial use of AZM in PA acute respiratory infections.

As AZM lacks serious direct antimicrobial activity on PA, the use of this molecule in the management of PA pneumonia could only be considered as an adjunct therapy, and the risk of drug interactions should be kept in mind. On one hand, recent papers warned of the potential antagonist effect of AZM on therapeutic benefits provided by tobramycin in CF patients with PA airway infections (Nick et al., 2014; Klingel et al., 2019). On the other hand, a synergism between QS inhibitors and antibiotics has been suggested in several *in vitro* studies, and paves the way for new trials combining AZM and anti-PA antibiotics (Vadekeetil et al., 2016; Bahari et al., 2017; Roudashti et al., 2017).

To go further, exposure to AZM could have impact on the human microbiome, with positive and negative effects that cannot be ignored. Indeed, there is emerging evidence that the lung microbiome exerts a major influence on the shaping of host immune response and on lung inflammation (Segal et al., 2013, 2016; Roquilly et al., 2019). It has recently been demonstrated that AZM modulates both lung microbiota and metabolome, thus increasing anti-inflammatory bacterial metabolites production (Segal et al., 2017). If modulation of the lung microbiome appeared beneficial in this trial, adverse impacts on microbiota should not be underestimated. Actually, long-term low-dose macrolide therapy in patients presenting bronchiectasis has been associated with significant changes in the oropharyngeal microbiota, and with significant increase in the carriage of transmissible macrolide resistance (Choo et al., 2018). In addition, ribosomal mutations have recently been described in PA from CF patients exposed to long-term macrolides treatment, suggesting acquired resistance to anti-virulence effects (Mustafa et al., 2017). It has also been shown that a single macrolide course is sufficient to sustainably alter murine intestinal microbiota (Ruiz et al., 2017). Thus, the use of an additional antibiotic as an adjunctive therapy should be considered with caution in the era of antibiotic resistance and antimicrobial stewardship. Recent advances in high throughput omic approaches should help understand the mechanistic basis and the extent of AZM action and could lead to rational for designing more efficient treatments.

## Author Contributions

AGL: writing original draft. JC, NC, AB, SC, KA, AR, and LC: writing review and editing. All authors contributed to the article and approved the submitted version.

### Conflict of Interest

The authors declare that the research was conducted in the absence of any commercial or financial relationships that could be construed as a potential conflict of interest.
